# Proinflammatory Cytokine Gene Polymorphisms in Bullous Pemphigoid

**DOI:** 10.3389/fimmu.2019.00636

**Published:** 2019-03-29

**Authors:** Pardis-Sadat Tabatabaei-Panah, Hamideh Moravvej, Zahra Sadaf, Hadis Babaei, Maryam Geranmayeh, Sedigheh Hajmanouchehri, Ahmad Karimi, Fatemeh Sajjadi, Fereshteh Arghand, Ralf J. Ludwig, Mareike Witte, Reza Akbarzadeh

**Affiliations:** ^1^Biology Department, East Tehran Branch, Islamic Azad University, Tehran, Iran; ^2^Skin Research Centre, Shahid Beheshti University of Medical Sciences, Tehran, Iran; ^3^Lübeck Institute of Experimental Dermatology, University of Lübeck, Lübeck, Germany; ^4^Department of Dermatology, University of Lübeck, Lübeck, Germany; ^5^Institute of Anatomy, University of Lübeck, Lübeck, Germany

**Keywords:** bullous pemphigoid, autoimmune disease, proinflammatory cytokines, gene polymorphism, gene expression

## Abstract

Bullous pemphigoid (BP) is a rare autoimmune skin blistering disease, characterized by the presence of autoantibodies against hemidesmosomal autoantigens. Cytokine expression is altered in BP patients, and several of these differently expressed cytokines, including IL-1α, IL-1β, IL-8, and TNF-α, contribute to disease pathogenesis. Since genetic polymorphisms in the genes of these cytokines might be implicated in susceptibility to BP disease, we aimed at testing this implication in susceptibility to BP in an Iranian cohort. Blood samples were collected from the subjects and genomic DNA was extracted. To detect the single nucleotide polymorphisms (SNPs), *IL-1*α (rs1800587), *IL-1*β (rs1143627, rs16944, rs1143634), *IL-8* (rs4073), and *TNF-*α (rs1799964, rs1800630, rs1799724, and rs361525) genes were genotyped in BP patients and healthy controls as well as *IL-8* (rs4073) in pemphigus vulgaris (PV) patients. Quantitative gene expression was evaluated by RT-PCR analysis. A significant difference was observed in the distribution of genotypes or alleles of *IL-8* SNP between the BP patients and controls. The A-allele of *IL-8* SNP is significantly more prevalent in the control individuals compared to the BP patient. To further validate this observation, we included PV patients as an additional control. Again, the A-allele of *IL-8* SNP is significantly more prevalent in the PV compared to the BP patients. While we observed a trend toward significant differences regarding alleles of *TNF-*α rs1799724 as well as alleles of *TNF-*α rs1799964, this difference was, however, not evident after correction for multiple analysis. There was no significant difference in all other studied SNPs. In contrast to *IL-1*α, *IL-1*β, and *TNF-*α, *IL-8* gene expression levels were significantly higher in the patients than that of controls. The minor allele in *IL-8* SNP might play a protective role in susceptibility to BP in Iranian patients. Although higher expression levels of *IL-8* gene was found in the patients compared with healthy controls, these levels, however, suggest no association with the examined polymorphism. Moreover, further investigation revealed an elevation in gene expression between wild and polymorphic genotypes of *IL-1*α rs1800587 and *TNF-*α rs361525 in the patient group and these SNPs are therefore associated with altering the levels of gene expression.

## Introduction

Bullous pemphigoid (BP) is a rare and difficult-to-treat autoimmune skin blistering disease characterized by the presence of an autoimmune response against type XVII collagen (COL17, BP180) and BP230, and presents with blisters and erythema, and/or urticarial plaques, where itch is clinically a leading symptom (1–4). Binding of autoantibodies to their antigens attenuate skin integrity and initiate a cascade of the inflammatory response (5). This response is mainly mediated by infiltration of inflammatory cells, deposition of complement, and activation of proteases and reactive oxygen species which finally result in blister formation (5). Cytokines, as regulators of inflammatory and immune reactions in the skin, contribute to inflammatory blister induction by recruitment of inflammatory cells as well as activation of resident cells (6).

Humoral immune-mediated mechanisms and cytokine involvement have been described in the pathogenesis of autoimmune blistering disorders (7). High levels of proinflammatory cytokines interleukin (IL) 1 alpha (IL-1α), IL-1β, IL-8, and TNF-α have been detected in the BP patients (8–10). IL-1α and IL-1β are two proinflammatory cytokines belong to the IL-1 family. IL-1 has central roles in infection, inflammation, and disease which the inflammatory process is initiated by IL-1α but multiplied and retained by both forms (11). Tumor necrosis factor- α (TNF- α) is another proinflammatory cytokine and acts as a central and multifunctional mediator of diverse cellular events (12). IL-1 and TNF- α can subsequently induce cytokines involved in leukocyte attraction, including IL-8 (13). IL-8 is a chemoattractant cytokine produced by a variety of cell types which contribute to a variety of proinflammatory activities (14).

Cytokine gene polymorphisms may influence the induction and release of cytokines, contribute to the disease-associated cytokine imbalance, and thereby affect the susceptibility to autoimmune diseases (15, 16). Gene polymorphisms of *IL-1*α, *IL-1*β, *IL-8*, and *TNF-*α cytokines have substantially been studied in several autoimmune diseases. To date, the contribution of cytokine polymorphisms in BP disease has not been investigated in great detail. One study investigated the cytokine polymorphisms in a Chinese cohort (17). One additional study focused on single polymorphism in *TNF-*α in Iranian population (18). In the Chinese cohort, a polymorphism in the *IL-1*β was identified in female patients only. In our study, in Iranian BP patients, we found no significant association of BP with *TNF* polymorphisms. With the exception of these two studies, no data is available about the association between proinflammatory cytokine polymorphisms and the development of BP. Since these polymorphisms might be associated with susceptibility to the diseases, the present study designed to examine the frequency of different polymorphisms in Iranian BP patients compared with healthy individuals. Due to different gene expression as a result of polymorphism, the RNA expression was evaluated in patients with different allelic variation and furthermore, compared with control subjects.

## Materials and Methods

### Study Population

Patients with BP disease were selected from Iranian patients attending the Shohada Tajrish, Loghman Hakim, and Razi hospitals in Tehran between 2013 and 2016. The patient group (*n* = 40) consisted of 12 males and 28 females, with an age ranging from 47 to 100 years with a mean age of 69.9 ± 2.01 years. BP was diagnosed based on clinical presentation, subepidermal blister on skin biopsy, compatible light microscopy findings, and positive direct immunofluorescence microscopy (DIF) data according to investigational assessment guidelines (19). Samples of the patients were collected immediately after diagnosis of BP and the peri-procedural assessment was performed by using the initial data analysis of the patients. Samples with a lack of demographic data or inappropriate processing of initial laboratory specimens which could bias the study are excluded. Relevant information was taken from all study participants such as suffering from autoimmune diseases, past history of BP, skin diseases, and heart diseases using questionnaires. Furthermore, 84 samples from patients with pemphigus vulgaris (PV) were used as control patients. Clinical presentation, as well as direct and indirect immunofluorescence, were performed for the diagnosis of PV. Furthermore, autoantibodies against desmoglein 3 were detected in all PV patients. Local ethics committee (Human Research Ethics Committee of Skin Research Center, Shahid Beheshti University of Medical Sciences) considered and approved the study protocol. Informed patient consent was obtained and the principles outlined in the Declaration of Helsinki was conducted in the study. Unrelated sex and age-matched healthy volunteers were also recruited without any evidence of previous BP. Control individuals (*n* = 40) included 20 males and 20 females, aged 46 to 92 years with a mean age of 64.7 ± 1.72 years.

### Single Nucleotide Polymorphism (SNP) Genotyping and Gene Expression Analysis

Whole blood was taken from the subjects and genomic DNA was extracted using isolation kit (DNGTM—Plus; SinaClon, Iran) according to the manufacturer's protocol. Genetic SNPs of *IL-1*α (rs1800587), *IL-1*β (rs1143627, rs16944, rs1143634), *IL-8* (rs4073), and *TNF-*α (rs1799964, rs1800630, rs1799724, and rs361525) genes were analyzed using primers and respective restriction endonucleases by PCR-based restricted fragment length polymorphism (PCR-RFLP) as described previously (20–25).

*IL-1*α, *IL-1*β, *IL-8*, and *TNF-*α gene expression was evaluated by quantitative RT-PCR, based on RNA extraction and cDNA synthesis from blood leucocytes of all subjects using RNX-Plus kit (SinaClon, Iran) according to the manufacturer's instructions. Expression of candidate genes, as well as housekeeping glyceraldehyde-3-phosphate dehydrogenase (GAPDH) gene, was analyzed by real-time RT-PCR using SYBR Green assays. Primer sequences and conditions were designed by ABI PCR equipment (Applied Biosystems, USA) except *GAPDH* which obtained from previously published sequences (26) (Supplementary Table 1).

### Statistical Analysis

Statistical analysis was carried out using the SPSS statistical software program or Prism software. *Post-hoc* power analysis of the present study was performed using G^*^Power (version 3.1) (http://www.gpower.hhu.de/). Genotype distribution, allele frequencies, and consistency with the Hardy–Weinberg equilibrium was analyzed using Chi-square (χ^2^) test. Linkage disequilibrium (LD) parameters delta coefficient (D′) and r squared (*r*^2^) of the population, as well as haplotype analysis, were calculated by comparisons among the SNPs using the Haploview software version 4.2 and SNPStats online software (http://bioinfo.iconcologia.net/snpstats/start.htm). The χ^2^ test, analysis of variance (ANOVA), or *t*-test are used to conduct a possible association between SNPs and clinical-demographic features. Logistic regression analysis was performed to predict the probability occurrence of BP. Shapiro–Wilk test was applied to verify the normality of data distribution and differences between groups, where Mann–Whitney *t*-test was used to analyze a non-Gaussian distribution. Data were expressed as median with interquartile range and a *P* < 0.05 was considered statistically significant. The *P*-value was corrected (*Pc*) for multiple analysis using the Bonferroni method.

## Results

### Study Population and Characterization

This study included 40 patients with BP and 40 age- and sex-matched controls. Sample size estimation and the statistical power calculation was conducted based on the reported prevalence of the variants. The sample size of the study is sufficient to reach 70% power at a significance level of 0.05 when considering the medium effect sizes of 0.3. There was no significant difference between patients and controls regarding age and gender (*P* > 0.05). Frequencies of *IL-8* (rs4073) and *TNF-*α (rs361525) SNPs in both patient and control groups, as well as the distribution of *IL-1*α (rs1800587), *IL-1*β (rs1143634), and *TNF-*α (rs1800630) in control individuals, deviated from Hardy–Weinberg equilibrium. Genotype frequencies of all other polymorphisms were within the equilibrium.

### Allele and Genotype Frequencies of Proinflammatory Cytokines

Gene variations of *IL-1*α, *IL-1*β, *IL-8*, and *TNF-*α were analyzed in BP patients compared to healthy controls to uncover whether these polymorphisms are associated with BP (Table 1). A significant association was found between the genotypes and alleles of our cases and controls in rs4073 of *IL-8*, where the polymorphic A-allele was significantly more present in the controls (42.5%) than to that of patient individuals (22.5%) (*Pc* = 0.01). Analysis of logistic regression indicated that the A-allele might be a protective factor for developing BP; the calculated odds ratio (OR) was 0.41 (95% confidence interval (CI): 0.18–0.89). In contrast to *IL-8*, we observed no significant difference in the distribution of genotypes or alleles of *IL-1*α and *IL-1*β SNPs between the BP patients and controls (*P* > 0.05). In *TNF-*α rs1799964, the frequency of C-allele was a two-fold increase in the control individuals (30%) compared to the patients (15%), which was significant (*P* = 0.02). Logistic regression analysis revealed that the minor C-allele at this locus of *TNF-*α might be protective for developing BP; the calculated odds ratio (OR) was 0.41 (95% confidence interval (CI): 0.18–0.89). However, this difference remains at the border of significance (*Pc* = 0.05). In *TNF-*α rs1799724, we found a significant difference in the distribution of genotypes (*P* = 0.03) and alleles (*P* = 0.04) as well as genotypes (*P* = 0.03) in *IL-1*α rs1800587, but this difference does not achieve the levels of significance after correction for multiple analysis. No significant association was found between our cases and controls in other SNPs of *TNF-*α (*P* > 0.05).

**Table 1:** Genotype and allele frequencies of *IL-1*α, *IL-1*β, *IL-8*, and *TNF-*α gene polymorphisms in Iranian patients with BP and respective controls.

**Gene**	**Genotype/allele**	**Patients with BP** **(*n* = 40)**	**Control subjects** **(*n* = 40)**	**OR (95% CI)**	***P***	***Pc***
*IL-1α* rs1800587	C/C	0.40	0.20	0.41 (0.19–0.91)	**0.03**	0.09
	C/T	0.45	0.70			
	T/T	0.15	0.10			
	C-allele	0.625	0.550	0.73 (0.39–1.37)	0.33	–
	T-allele	0.375	0.450			
*IL-1β* rs16944	C/C	0.35	0.30	0.71 (0.36–1.38)	0.31	–
	C/T	0.55	0.50			
	T/T	0.10	0.20			
	C-allele	0.625	0.550	0.73 (0.39–1.37)	0.33	–
	T-allele	0.375	0.450			
*IL-1β* rs1143627	T/T	0.35	0.30	0.91 (0.51–1.63)	0.76	–
	T/C	0.40	0.45			
	C/C	0.25	0.25			
	T-allele	0.550	0.525	0.90 (0.48–1.68)	0.75	–
	C–allele	0.450	0.475			
*IL-1β* rs1143634	G/G	0.55	0.40	1.40 (0.62–3.15)	0.41	–
	G/A	0.40	0.60			
	A/A	0.05	0			
	G-allele	0.750	0.700	0.77 (0.38–1.56)	0.47	–
	A-allele	0.250	0.300			
*IL-8* rs4073	T/T	0.70	0.25	0.40 (0.20–0.82)	**0.01**	**0.03**
	T/A	0.15	0.65			
	A/A	0.15	0.10			
	T-allele	0.775	0.575	0.39 (0.19–0.78)	**0.008**	**0.01**
	A-allele	0.225	0.425			
*TNF-α* rs1799964	T/T	0.75	0.55	0.49 (0.24–1.00)	0.05	–
	T/C	0.20	0.30			
	C/C	0.05	0.15			
	T-allele	0.850	0.700	0.41 (0.18–0.89)	**0.02**	0.05
	C-allele	0.150	0.300			
*TNF-α* rs1800630	C/C	0.75	0.60	0.55 (0.27–1.12)	0.10	–
	C/A	0.20	0.25			
	A/A	0.05	0.15			
	C-allele	0.850	0.725	0.46 (0.21–1.02)	0.05	–
	A-allele	0.150	0.275			
*TNF-α* rs1799724	C/C	0.70	0.90	3.85 (1.12–13.2)	**0.03**	0.09
	C/T	0.30	0.10			
	T/T	0	0			
	C-allele	0.850	0.950	3.35 (1.03–10.8)	**0.04**	0.08
	T-allele	0.150	0.050			
*TNF-α* rs361525	G/G	0.30	0.15	0.41 (0.13–1.23)	0.11	–
	G/A	0.70	0.85			
	A/A	0	0			
	G-allele	0.650	0.575	0.72 (0.38–1.37)	0.33	–
	A-allele	0.350	0.425			

*P-values (P) were performed based on the logistic regression test and a value of < 0.05 was considered statistically significant. BP, bullous pemphigoid; OR, odds ratio; CI, confidence interval; n, number; Pc, Corrected P-value. Statistically significant P-values are shown in bold*.

Given the significant association between BP patients and respective healthy controls in *IL-8* rs4073 and in order to uncover whether the observed association is BP-specific, this polymorphism was likewise analyzed in 84 PV patients (Table 2). The genotypes and alleles were similarly distributed between PV patients and healthy controls; the calculated odds ratio (OR) by logistic regression analysis for A-allele was 1.06 [95% confidence interval (CI): 0.62–1.82]. However, when comparing genotypes and alleles of PV to BP patients, the polymorphic A-allele was significantly more present in PV as compared to BP patients; odds ratio (OR) for A-allele was 0.58 [95% confidence interval (CI): 0.34–0.99]. Although this significance for allele distribution disappears after correction for multiple analysis, it remains significant for genotype analysis.

**Table 2:** Comparison of genotype and allele frequencies of *IL-8* rs4073 gene polymorphism in PV Iranian patients with healthy control subjects as well as BP patients.

**Gene**	**Genotype/allele**	**Patients with PV** **(*n* = 84)**	**Patients with BP** **(*n* = 40)**	**Control subjects** **(n = 40)**	**OR (95% CI)^†^**	**OR (95% CI)^‡^**	P^*^	P^**^
*IL-8* rs4073	T/T	0.32	0.70	0.25	1.07 (0.61–1.87)	2.35 (1.32–4.21)	0.81	**0.004**^#^
	T/A	0.48	0.15	0.65				
	A/A	0.20	0.15	0.10				
	T-allele	0.56	0.775	0.575	1.06 (0.62–1.82)	0.58 (0.34–0.99)	0.81	**0.04**^#^
	A-allele	0.44	0.225	0.425				

*P values (P) were performed based on the logistic regression test and a value of < 0.05 was considered statistically significant. BP, pemphigus vulgaris; OR, odds ratio; CI, confidence interval; n, number; Pc, Corrected P-value*.

* PV vs. control;

** *PV vs. BP*.

† PV vs. Control;

‡ *PV vs. BP*.

# *Genotype Pc: 0.012; Allele Pc: 0.12. Statistically significant P-values are shown in bold*.

### Linkage Disequilibrium and Haplotype Frequencies

**Figure 1:**
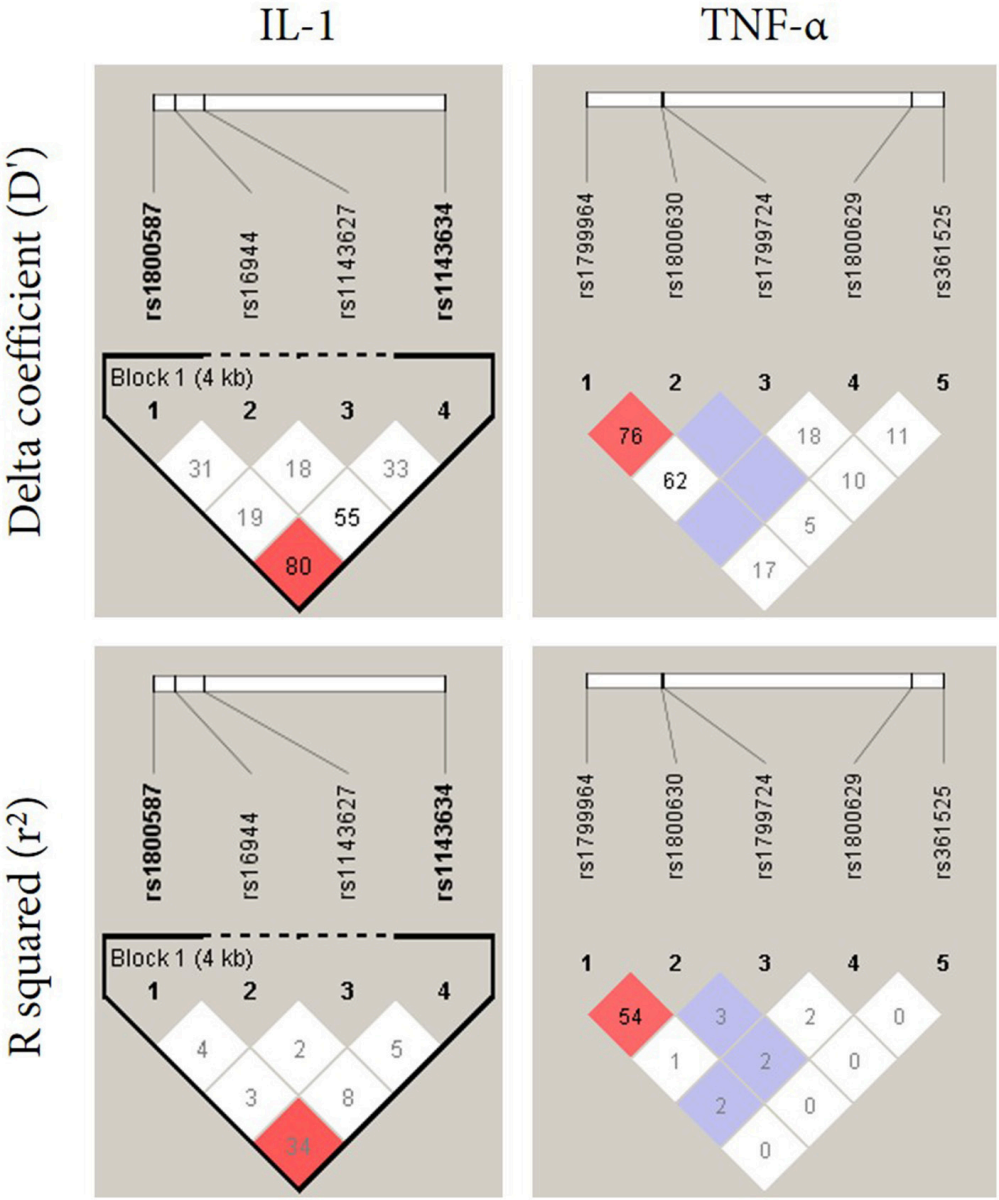
Linkage disequilibrium pattern of the genomic region in chromosome 2 and 6 located between SNPs *IL-1*α and *IL-1*β (rs1800587, rs1143627, rs16944, and rs1143634) and *TNF-*α (rs1799964, rs1800630, rs1799724, rs1800629, and rs361525), respectively.

Linkage disequilibrium (LD) was performed in order to investigate the relationship between polymorphisms in *IL-1* and *TNF-*α (Figure 1). The LD analysis of the genotyped SNPs in our samples suggested that two SNPs (rs1800587 and rs1143634) in *IL-1* are in strong linkage disequilibrium (*D*′ = 0.80*, LOD* = 2.94) (Figure 1, left panel). No correlation was found between polymorphisms in *TNF-*α (Figure 1, right panel). Haplotype frequencies were compared between *IL-1* and *TNF-*α SNPs and their association with BP which is summarized in Table 3. The *P*-values for individual and global haplotype score tests did not indicate a significant difference in haplotype frequency profiles between cases and controls (*P* > 0.05).

**Table 3:** Haplotype patterns with their frequencies in the population.

***IL-1α*** **and** ***IL-1β*** **(rs1800587, rs1143627, rs16944, and rs1143634)**				***TNF-α*** **(rs1799964, rs1800630, rs1799724, rs1800629, and rs361525)**			
**Haplotype**	**Frequency**	**χ**^**2**^	***P*-value**	**Haplotype**	**Frequency**	**χ**^2^	***P*-value**
CTTG	0.22	0.07	0.78	TCCGG	0.22	2.73	0.09
CCCG	0.14	0.01	0.89	TCCGA	0.14	0.09	0.76
TCCA	0.13	0.03	0.85	CACGG	0.13	0.09	0.75
CCTG	0.11	0.78	0.37	CACGA	0.11	1.59	0.20
TCTG	0.09	0.08	0.77	TACGG	0.09	2.98	0.08
CTCG	0.07	0.15	0.69	TCTGA	0.07	0.06	0.79
Global haplotype association *p*-value			0.2	Global haplotype association *p*-value			0.08

*Haplotypes with a frequency < 5% are not listed. P < 0.05 was considered statistically significant*.

### Genotype Association of BP Patients With Clinical and Demographic Characteristics

Clinical and demographical features were analyzed for a possible association between patients with wildtype and polymorphic genotypes in which the *P*-values are shown in Table 4. Of note, patients with polymorphic genotypes in *TNF-*α rs1799964 (T/C+C/C) and rs1800630 (C/A+A/A) SNPs were significantly older than patients with wild-type genotypes (T/T and C/C, respectively) (*P* = 0.001). Similarly, in these SNPs patients with polymorphic genotypes developed the BP disease in older ages (*P* = 0.002) and were suffering from heart diseases and focal infection. Furthermore, patients with polymorphic genotypes in *TNF-*α rs1799724 (C/T +T/T) and rs361525 (G/A+A/A) SNPs had anemia, depression, or other skin diseases. In the case of *IL-1*β rs1800587, comparison of BP patients carrying wild genotype (C/C) against those with polymorphic genotypes (C/T+T/T) demonstrated that the majority of patients with polymorphic genotypes were suffering heart disorders (*P* = 0.005). Comparison of cytokine gene polymorphisms in male/female subjects of patients suggested that the polymorphic genotypes of *IL-1*β rs1143627 and rs1143634, as well as *TNF-*α rs1799964 and rs1800630, were significantly more frequent in female patients than in male subjects.

**Table 4:** Association of clinical characteristics and demographic data in BP patient with polymorphic and wild genotypes in *IL-1*α, *IL-1*β, *IL-8*, and *TNF-*α gene polymorphisms.

	***P-*****value**								
	***IL-1α***	***IL-1β***			***IL-8***	***TNF-α***		
Characteristics	rs1800587	rs16944	rs1143627	rs1143634	rs4073	rs1799964	rs1800630	rs1799724	rs361525
Age (years)	0.26	0.50	0.17	0.63	0.95	**0.001**	**0.001**	0.35	0.43
Disease duration (years)	0.53	0.17	0.90	0.90	0.10	0.17	0.17	0.68	0.87
Age of onset (years)	0.19	0.31	0.31	0.55	0.76	**0.002**	**0.002**	0.44	0.30
Gender, male/female	0.57	0.12	**0.000**	**0.02**	0.24	0.99	**0.01**	0.07	0.07
Autoimmune diseases	0.57	0.88	0.42	0.34	0.99	0.42	0.42	0.76	0.76
Familial history of BP	0.23	0.99	0.40	0.73	0.99	0.99	0.40	0.34	0.34
Heart diseases	**0.01**	0.05	0.46	0.14	0.17	**0.04**	**0.02**	0.16	1.00
Hypertension	0.10	0.44	0.70	0.28	0.19	0.70	0.70	0.88	0.11
Focal infection	0.57	0.19	0.42	0.34	0.76	**0.02**	**0.01**	0.07	0.07
Skin Diseases	0.13	0.06	0.20	0.40	0.43	0.67	0.67	0.42	**0.01**
Anemia	0.29	0.11	1.00	0.23	0.40	1.00	1.00	**0.02**	0.39
Stress	0.43	0.13	0.71	0.18	0.67	0.71	0.71	0.33	0.07
Depression	0.13	0.06	0.67	0.40	0.43	0.21	0.20	**0.01**	0.42

*P-values (P) were performed based on the chi-square or t-test and a value of < 0.05 was considered statistically significant. Statistically significant P-values are shown in bold*.

### Gene Expression in BP Disease

**Figure 2:**
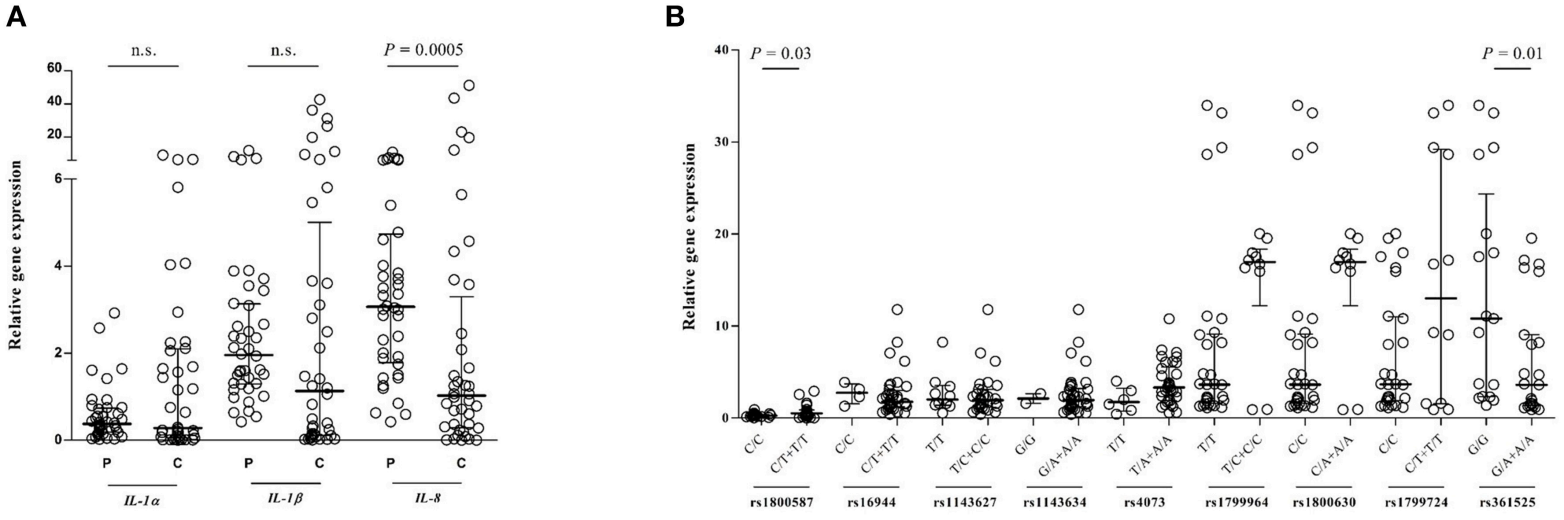
Quantitative real-time RT-PCR analysis of *IL-1*α, *IL-1*β, and *IL-8* RNA expression of the patients with BP or control individuals. Comparison of candidate gene expressions/*GAPDH* ratio is shown between patient and control subjects **(A)** as well as between wild and polymorphic genotypes in the patient group **(B)**, expressed as median with interquartile range and were compared based on the Mann-Whitney U-test. n.s., non-significant.

To investigate whether BP disease influences gene expression, a quantitative analysis of the *IL-1*α, *IL-1*β, and *IL-8* genes were performed which are shown in Figure 2A. Previously, we could show that the RNA expression levels of *TNF-*α elevate in circulating leukocytes obtained from patients with BP compared with control subjects (18). Although the gene expression of *IL-1*β was higher in the controls than in patients, it did not reach the levels of significance (*IL-1*β/*GAPDH*: 1.95 [0.42–11.7] vs. 1.13 [0.006–42.5], *P* = 0.07). The *IL-8* gene was expressed significantly higher in the patient subjects than to that of control (*IL-8*/*GAPDH*: 3.06 [0.42–10.7] vs. 1.02 [0.001–51.1], *P* = 0.0005). There is no significant difference between patients and controls in *IL-1*α gene expression. Further investigation revealed an alteration in gene expression between wild and polymorphic genotypes of *IL-1*α rs1800587 (*P* = 0.03) and *TNF-*α rs361525 (*P* = 0.01) in the patient group and these SNPs are therefore associated with altering the levels of gene expression (Figure 2B).

## Discussion

Cytokines such as IL-1, IL-8, and TNF-α are key pro-inflammatory cytokines and play an essential role in inflammatory response through cell activation and triggering a cytokine cascade (27). Despite elevated levels of these cytokines have been demonstrated in BP, the obvious role of cytokines remains controversial. Since altered expression of cytokines could affect the pathogenesis of BP, functional polymorphisms in cytokine genes may alter cytokine production and its biological balance which in terms results in higher susceptibility or severity of BP disease. Lack of sufficient data about any possible effect of gene variations on susceptibility to BP disease in different ethnicity indicates a necessity for further investigations. This is the first study to the best of our knowledge, which investigates the association of alleles and genotypes of proinflammatory susceptible genes *IL-1*α, *IL-1*β, *IL-8*, and *TNF-*α with the risk of BP as well as the influence of these variations on cytokine gene expression.

IL-1α and IL-1β cytokines stimulate the release of chemotactic cytokines such as IL-8, either directly or in synergy with TNF-α (28, 29) and are involved in the pathologies of several skin diseases including psoriasis, cutaneous lupus erythematosus, atopic dermatitis, and autoimmune blistering diseases (11, 30). IL-1α and IL-1β, mainly produced by the keratinocytes in the skin, as well as TNF-α are key inflammatory cytokines in inflammatory skin diseases such as dermatomyositis and pemphigus (31), as well as experimental pemphigoid disease (32, 33). IL-1β stimulates the inflammatory signals by inducing other cytokines such as TNF-α and IL-8 (13). In our cohort study, we discovered an association between polymorphism of *IL-8* cytokine in BP patients compared to the controls. There is disagreement in the results reported for *IL-8* rs4073 in Iranian populations with inflammatory diseases. While a study has shown no association between the occurrence of chronic periodontitis (34), another investigation was observed a significant difference for this polymorphism in the same disease (35), which could be due to the diverse ethnicity of the Iranian population. In agreement with the later results of Iranian population, our data indicate that the minor allele is protective for developing the BP disease. Of note, analysis of PV patients revealed that this association is specific for BP because the allele distribution was indicial between healthy controls and PV patients.

However, our observations for *IL-8* rs4073 is in contrast to data reported by a publication from China which investigated cytokine gene polymorphisms in a Chinese population (17). In this study, we found similar allele and genotype distribution of all studied SNPs in *IL-1*α and *IL-1*β genes. Except for *IL-1*β (rs1143627) (36), these findings are in line with observations of Iranian investigations on autoimmune diseases (37, 38). Among four studied polymorphisms in *TNF-*α, our observation revealed that the prevalence of minor allele in rs1799724 is significantly higher in the patients than controls, confirming previously published date from Iran in inflammatory disease (39). However, this significance disappeared after correction for multiple analysis. Similar to reported findings from Iran on autoimmune or inflammatory diseases, no significant differences were observed in our cohort study in all other SNPs in *TNF-*α (40–42).

There are controversies regarding the concentration of IL-1 in the blister fluid or sera of BP patients (43, 44). Nevertheless, since there is a relationship between IL-1β, IL-8, and TNF-α levels and the number of skin lesions in the patients, they might play a pathogenic role in BP disease (8, 45). Furthermore, blocking of pathogenic effect of rabbit IgG by using neutralizing IL-1 antibodies in an experimental mouse model of BP suggest a critical role of this cytokine in autoimmune blistering diseases (46). Genetic polymorphisms which are located in genes encoding for susceptibility factors may contribute to the gene expression or protein function and in turn to the disease predisposition in certain individuals. Our previous data indicate that the serum or plasma levels of TNF-α are significantly higher in the patients with BP or acute myocardial infarction to that of controls (18, 47). We could also show that the −308A allele is associated with elevated levels of *TNF-*α mRNA and protein expression in the patients which in turn might be contributed in increased transcription levels of *TNF-*α gene (47). There is also evidence that the −238G, −863A, and −1031C alleles influence the up-regulation levels of *TNF-*α expression in individuals (48, 49). In contrast, the −238A and −857T alleles are associated with lower levels or inhibition of *TNF-*α expression and transcription (50, 51). In other studies, it has been shown a higher serum level of IL-1β in patients with Alzheimer disease who carry −511T/T genotype (52). The SNPs in the *IL-8* gene (-251A/T and +781C/T) have been shown to be associated with IL-8 production or protein expression both *in vivo* and *in vitro* (53, 54). Since the influence of the BP disease on cytokine gene expression as well as the effect of the SNPs in alteration of gene expression levels are still controversial, a quantitative analysis of the *IL-1*α, *IL-1*β, and *IL-8* genes was performed. In agreement to previous investigations, the *IL-8* gene was expressed significantly higher in the patients than to that of control. However, these expression levels of *IL-8* gene suggest no association with the examined polymorphisms and comparable levels were found in wild-type and polymorphic genotypes. In contrast, further investigation revealed that variations of *IL-1*α rs1800587 and *TNF-*α rs361525 might contribute to altering the levels of gene expression.

The data presented herein has to be interpreted considering the limitations of our study. First, our study has a power of 70% to detect assumed differences of 30%. Hence, we may have missed significant associations due to the relatively low power of the study. However, nothing had been known about the investigated polymorphisms. Hence, our data now allows for a precise sample size calculation for future studies. In addition, despite this limitation, we identified one significant association. Second, the diagnosis of BP, like in the BLISTER study (55) was based on clinical presentation and DIF. Subsequent serological analysis of the BLISTER Study confirmed BP diagnosis in almost 90% of the cases (56). Hence, a maximum of 10% of our patient population may have had an alternative diagnosis, such as p200 pemphigoid, mucous membrane pemphigoid or epidermolysis bullosa acquisita. Third, genetic association studies cannot provide functional insights into the identified associated polymorphisms. However, these genetic studies may be used as a guide for further studies, addressing the functional impact of the newly identified associated gene polymorphisms.

In conclusion, our findings suggest that the minor allele in *IL-8* SNP might play a protective role in susceptibility to BP in Iranian patients. Possible protective influences of *IL-8* (rs4073) polymorphism against auto-inflammatory disorders might be helpful toward developing potential therapeutic strategies that intervene with the functions of inflammatory cytokine IL-8 in patients with BP. Given that this association was not observed in PV patients, this would also allow a more specific treatment of different pemphigus and pemphigoid diseases.

## Data Availability

All datasets generated for this study are included in the manuscript and/or the Supplementary Files.

## Author Contributions

RA, P-ST-P, ZS, HB, MG, SH, AK, FS, and FA participated in the experiments. HM, RL, and MW provided the samples and performed the diagnosis of the disease. P-ST-P and RA designed the study and performed data analysis. RA wrote the manuscript and RL revised the manuscript.

### Conflict of Interest Statement

The authors declare that the research was conducted in the absence of any commercial or financial relationships that could be construed as a potential conflict of interest.

## Supplementary Material

Supplementary Table 1 PCR condition and primer pairs for Quantitative RT-PCR of *IL-1*α, *IL-1*β, *IL-8, TNF-*α, and *GAPDH* gene expression levels.
