# Supplementary material for: Proinflammatory Cytokine Gene Polymorphisms in Bullous Pemphigoid
Source: Front Immunol. 2019 Mar 29;10:636. doi: 10.3389/fimmu.2019.00636 (PMC6455081; doi:10.3389/fimmu.2019.00636)
Supplement: Supplementary Table 1 — PCR condition and primer pairs for Quantitative RT-PCR of IL-1α, IL-1β, IL-8, TNF-α, and GAPDH gene expression levels. [file Table_1.DOCX]

**sTable 1**

| Gene | Direction | Sequence | Annealing temperature (°C) |
| --- | --- | --- | --- |
| IL-1α | Forward  Reverse | 5′-ATGTGACTGCCCAAGATGAA-3′  5′-CCCAGAAGAAGAGGAGGTTG-3′ | 59 |
| *IL-1β* | Forward  Reverse | 5'-CAGATGAAGTGCTCCTTCCA-3′  5'-ACCAGCATCTTCCTCAGCTT-3' | 59 |
| *IL-8* | Forward  Reverse | 5'-AGGACAAGAGCCAGGAAGAA-3′  5'-ACTGCACCTTCACACAGAGC-3' | 59 |
| *GAPDH* | Forward  Reverse | 5'-ATGGAGAAGGCTGGGGCT-3′  5'-ATCTTGAGGCTGTTGTCATACTTCTC-3′ | 62 |
